# Synthesis of strontium oxide-zinc oxide nanocomposites by Co-precipitation method and its application for degradation of malachite green dye under direct sunlight

**DOI:** 10.1016/j.heliyon.2023.e20824

**Published:** 2023-10-11

**Authors:** Govindharaj Anandhakumari, Palanisamy Jayabal, Athinarayanan Balasankar, Subramaniyan Ramasundaram, Tae Hwan Oh, Kanakaraj Aruchamy, Parashuram Kallem, Veerababu Polisetti

**Affiliations:** a Department of Physics, Gobi Arts & Science College, Gobichettipalayam, Erode, Tamilnadu-638 453, India; b School of Chemical Engineering, Yeungnam University, Gyeongsan 38541, South Korea; c Wallenberg Wood Science Center, Department of Fibre and Polymer Technology, School of Engineering Sciences in Chemistry, Biotechnology and Health, KTH Royal Institute of Technology, SE−100 44 Stockholm, Sweden; d Department of Environmental and Public Health, College of Health Sciences, Abu Dhabi University, Abu Dhabi, P.O. Box 59911, United Arab Emirates

**Keywords:** Strontium oxide, Zinc oxide, Nanocomposites, Sunlight, Photocatalyst, Dye degradation and malachite green

## Abstract

Photocatalysts workable under direct sunlight are the safe and cost-effective option for water purification. The nanocomposites of strontium oxide and zinc oxide (SZ NCs) were synthesized using coprecipitation method. The respective precursors of SZ NCs were subjected to alkaline hydrolysis and subsequently thermally treated to yield SZ NCs. The SZ NCs with different ZnO composition was synthesized by varying the concentration of ZnO precursor from 0.2 to 1 M. The structural properties of SZ NCs evaluated using X-Ray diffraction (XRD), Thermogravimetric analysis (TGA), and Differential thermal analysis DTA). The optical properties of SZ NCs studied using ultraviolet–visible (UV–Vis) spectroscopic study. The trend observed in the intensity of XRD peaks indicated the occurrence of Zn doping in the crystalline lattice of SrO and the formation of SrO–ZnO composite. Upon incorporation of 1 M of ZnO precursor, the grain size of the SrO was decreased from 49.3 to 27.6 nm. The weight loss in the thermal analysis indicates the removal of carbonates from the sample upon heating and shows the formation of an oxide structure. UV–Vis spectra confirmed that the presence of SrO enhanced the sunlight absorption of SZ NCs. The increase in the composition of ZnO precursors increased the bandgap of SrO (2.09 eV) to the level of ZnO (3.14 eV). SZ NCs exhibited heterostructure morphology, where the nanosized domains with varying shapes (layered and rod-like) were observed. Under direct sunlight conditions, SZ NCs prepared using 1 M/0.6 M of SrO/ZnO precursors exhibited 15–20 % higher photocatalytic efficiency than neat SrO and ZnO. In precise, 1 mg of this SZ NC was degraded 98 % of malachite green dye dissolved in water (10 ppm) under direct sunlight. Additionally, the thermal stability results showed that 18 % decomposition was obtained due to the degradation impurities in SrO/ZnO catalysts and the XRD results revealed that no structural change is obtained in SrO/ZnO photocatalysts after stability test. The SZ NCs can be effectively used as safe and economic sunlight photocatalysts for water purification in remote areas without the electricity.

## 1 Introduction

In recent years, metal oxide based semiconducting nanomaterials have been recognized as the potential photooxidation agents because of their unique physical and chemical properties [1]. They are noted to have the ability to mineralize the harmful organic compounds present in the effluent discharged from the textile, leather, paint, and pharmaceutical industries [[2], [3], [4], [5], [6]]. When irradiated with the light source with appropriate energy, the electrons in the surface of these metal oxide semiconductors excited and led to the generation of electron and hole pairs. The photo-generated electron-hole pair in turn reacts with water and O_2_ in air and renders the strong reactive oxygen species [[7], [8], [9]]. In principle, these metal oxide semiconductors serve as heterogeneous photocatalysts [10] that can photo oxidizes the organic compounds dissolved in water. Thus, by virtue, these materials were considered as most suitable for removing effluent from secondary waste-water treatment, which is free from settleable and floatable solid impurities [11]. Titanium dioxide (TiO_2_) and zinc oxide (ZnO) are commonly used wide bandgap (3.2 eV) metal oxide semiconductor photocatalysts. Because of their chemical stability, abundance, non-toxicity, and low cost, TiO_2_ and ZnO are considered as suitable for practical application. Moreover, in photocatalysis, ZnO can exhibit a high potential because of its direct bandgap, anisotropic crystal growth, and rapid charge carrier mobility. The lifetime (>10 s) and mobility (200–300 cm^2^ V^−1^ s^−1^) of an electron in ZnO are higher than life time (75–350 ps) and mobility (0.1–4.0 cm^2^ V^−1^ s^−1^) of TiO_2_. Due to the higher mobility, the charge carriers easily migrate to the surface of ZnO upon light absorption thereby generating electron-hole pairs. These electron-hole pairs play an effective role in the photocatalysis reaction [12,13].

For environmental applications, the potential of ZnO photocatalysis has been not realized because ZnO is more amenable to acid/alkaline corrosion, and encounters rapid recombination of photo-generated electron hole pairs. Similar to TiO_2_, due to its large band gap (3.2 eV) ZnO also work under harmful ultraviolet light, constituting only 4 % in the sunlight. ZnO photocatalyst also suffers due to low recyclability [14]. Surface modification and formation composite with other metal oxides composites were reported as useful for addressing the issues pertaining to ZnO. The corrosion resistance of ZnO was enhanced for forming a thin layer of SiO_2_ or TiO_2_ [15,16]. Heterojunctions formed with low band gap semiconductors such as CdS, BiVO_4_, and Ag_3_PO_4_ were extended lifetime of charge carriers by preventing their rapid recombination and also imparted the visible light photocatalytic activity [15]. Visible light constitutes 43 % of solar radiation. Coupling of ZnO with high Eg (5.5–5.9 eV) semiconductor, strontium oxide (SrO) also proved as beneficial for increasing the lifetime of charge carriers [17,18] and as well as the utilization of solar radiation. In the band gap posting of ZnO and SrO, location of the valence band (VB) of ZnO is situated in between the VB and conduction band (CB) of SrO. The CB of ZnO is positioned above the SrO. Therefore, when excited with visible light of sufficient photon energy, the electron in the VB of ZnO moves to the CB of SrO while holes migrate to its VB [18]. The effective charge separation and possibility for the utilization of visible light make ZnO–SrO heterojunction as a potential option for environmental photocatalysis under direct sunlight. Low band gap SrO (∼1.8–2 eV) nanoparticles and nanorods showing light absorption at visible region were also reported [[19], [20], [21]].

Harish et al. prepared the ZnO–SrO nanocomposites by the one-pot hydrothermal method. In the ZnO–SrO nanocomposites, the ZnO–SrO heterojunction was formed due to the coating of SrO nanoparticles on the surface of ZnO. When studied using methylene blue dye as a target organic pollutant, this composite exhibited a 9-fold higher photocatalytic under visible light [22]. Rizwan et al. reported the improved photocatalytic activity of ZnO by Mn and Sr doping. The formation of intermediate state and hindered recombination leads to higher photocatalytic activity in the doped ZnO system [23,24]. The synergistic effect of binary composites of SnO_2_ and Mn_2_O_3_ composites towards the utilization of sun light and reduced recombination reported by the Rabia et al. [25]. ZnO–SrO composites were also prepared by solid-state sintering at a temperature above 1000 °C. The resultant composites were explored for non-photocatalytic application. The ZnO–SrO nanocomposites prepared by ZuO et al. by sintering precursors at 1050 °C was used as the catalyst for the depolymerization of poly(ethylene terephthalate) under microwave radiation. The hydrolytic depolymerization rate of 93.13 % was achieved when poly(ethylene terephthalate) was treated with 0.5 % of catalyst [26]. Rohini et al. prepared ZnO–SrO by sintering at 1250 °C, the SrO addition was enhanced the performance of ZnO varistors components used in arrestors meant for protecting the electrical equipment from external and internal overvoltage [27]. The economic route for the synthesis of ZnO is alkaline hydrolysis of the suitable precursor at ambient temperature conditions [28]. SrO was synthesized by alkaline hydrolysis [29,30] and also by using Ocimum sanctum leaf extract as a reducing agent [31]. The use of alkaline hydrolysis or coprecipitation method will be crucial for preparing inexpensive ZnO–SrO nanocomposite active under direct sunlight.

In the present work, SrO–ZnO nanocomposites (SZ NCs) were synthesized using the alkaline coprecipitation method. To the best of our knowledge, there was no such taken. Also, the performance of SZ NCs under direct sunlight is not yet reported elsewhere. The ZnO–SrO NCs with varying Zn content were synthesized. Zinc acetate dihydrate and strontium acetate hemihydrates were used as precursors for ZnO and SrO, respectively. The resultant NCs were characterized using X-ray diffraction (XRD) analysis, Field emission scanning electron microscope (FESEM), Ultraviolet–Visible (UV–Vis), and infrared spectroscopic studies. The photocatalytic properties of these NCs were evaluated under direct sunlight. The basic dye, malachite green (MG) was used as a model organic pollutant. For comparison, ZnO and SrO nanoparticles were synthesized separately and subjected to characterization identical to the ZnO–SrO NCs.

## 2 Experimental

### 2.1 Materials

Strontium acetate hemihydrates (Sr(CH_3_COO)_2_.5H_2_O, 98 %)), Zinc acetate hexahydrate (Zn(CH_3_COO)_2_.2H_2_O, 98 %), Sodium hydroxide (NaOH, 98 %), Malachite green (C_23_H_25_ClN_2_ 98 %), and ethanol (C₂H₆O, 99 %) were procured from Merck, India, and used as received. Deionized water (D.I.) was prepared in the laboratory and used wherever required during the experiments.

### 2.2 Synthesis of SrO and ZnO nanoparticles

Strontium oxide nanoparticles were synthesized by using the co-precipitation Method. In a typical synthesis, 0.5 M of strontium acetate hemihydrates was gradually added to 50 ml of ethanol under stirring. The 1 M sodium hydroxide solution (10 ml) was separately prepared and added dropwise to the mixture at room temperature for an hour (h). The pale white slurry was formed, and it was washed several times with D.I. water and ethanol. The washed precipitate was dried in a hot air oven at 60 °C for an h. Then, it was annealed at 800 °C in a muffle furnace for 2 h. After annealing, SrO NPs powders were collected and stored in airtight vials. The ZnO was also synthesized using the identical method, where, 0.5 M of zinc acetate dehydrate was taken instead of strontium acetate hemihydrates and it was annealed at 400 °C.

### 2.3 Synthesis of SrO–ZnO NCs

**Fig. 1:**
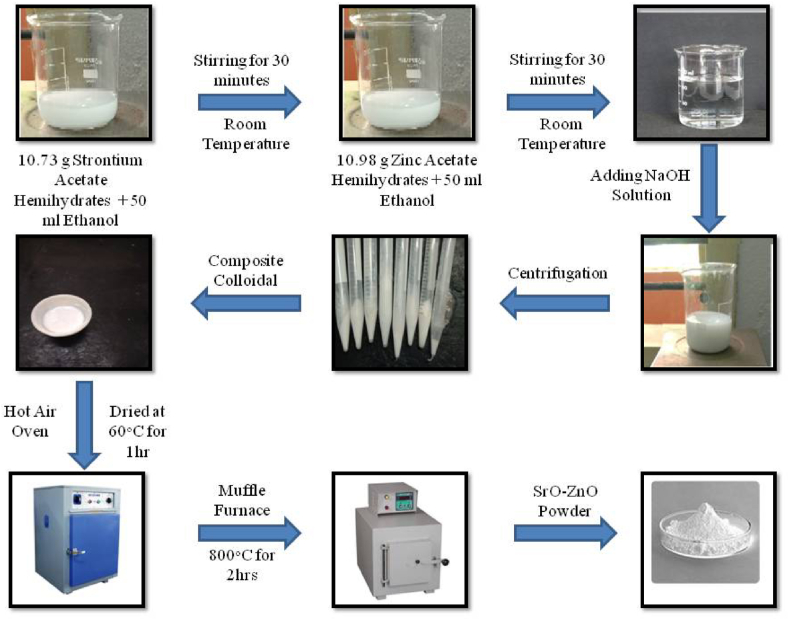
Schematic of wet chemical method followed to synthesize SrO–ZnO nanocomposites.

The schematic of steps followed to synthesize SZ NCs is shown in Fig. 1. Strontium acetate hemihydrates (1 M, 10.73 g) and zinc acetate dihydrate (1 M, 10.98 g) were gradually mixed in 100 ml of ethanol solutions under continuous stirring. Then, 10 ml aqueous solution and sodium hydroxide (1 M) were added drop-wise to the above mixture and stirred for 2 h. The obtained precipitate was washed with D.I. water and absolute ethanol. Washed precipitate was dried at 60 °C for 1 h, and annealed at 800 °C for 2 h. The Zn content in the ZnO–SrO NCs was varied by changing the concentration of zinc acetate dihydrate (0.2, 0.4, 0.6, 0.8, and 1 M). The samples were coded as ZnO–SrO-X, where X = the concentration of zinc acetate dihydrate used.

### 2.4 Characterization

The crystal structure and grain size of SZ NCs were studied by the PW3071 X-ray diffractometer. The Cu–K α radiation was used. The surface morphology was observed using Quanta 200F FESEM coupled with EDAX, and operated at the accelerating voltage of 30 KV. Electronic absorption and band-gap of the SZ NCs were obtained from the UV–Vis spectra using a JASCO V-750 UV–Vis spectrophotometer in diffused reflectance method. The functional group present in the SZ NCs was measured using PerkinElmer Spectrum two spectrometer with diamond ATR in the mid-IR range (4000-400 cm^−1^).

### 2.5 Photocatalysis experiments

The photocatalytic activity of SZ NCs was evaluated using 10 ppm of an aqueous solution of MG dye under direct sunlight. All photocatalysis experiments were performed at the premises of Gobi Arts & Science College, Gobichettipalayam, Tamilnadu, India. In 250 ml Erlenmeyer ﬂask, 1 mg ZnO–SrO NCs and 100 ml of 10 ppm MG solution were taken. To establish adsorption-desorption equilibrium, the MG solution was stirred for 30 min under dark conditions. Then, the contents of MG solution were exposed to direct sunlight in clear sky condition between 11 and 2 p.m. An aliquot of 5 ml was withdrawn at every 20 min time interval. The collected aliquot was centrifuged, and the supernatant was collected to evaluate the changes in MG concentration as a function of photocatalysis time. Withdrawn aliquots were analyzed using a JASCO V-750 UV–Visible spectrophotometer. The electronic absorption maxima of MG at 617 nm were taken into consideration. The changes in concentration of MG dye were calculated using the Lambert-Beer law. The dye degradation efficiency was determined using the following equation.

$$Dye\;degradion\;Effiency\;\left(\%\right)=\frac{C-C_{0}}{C_{0}}\;X\;100$$

Where, C_0=_Initial absorbance of the dye solution; and C = Absorbance of the dye solution after catalytic degradation at regular intervals time. For the repeatability, the photocatalytic measurements were repeated twice and the average data plotted is shown in Fig. 13.

## 3 Results and discussion

### 3.1 Crystalline structure SrO–ZnO nanocomposites

**Fig. 2:**
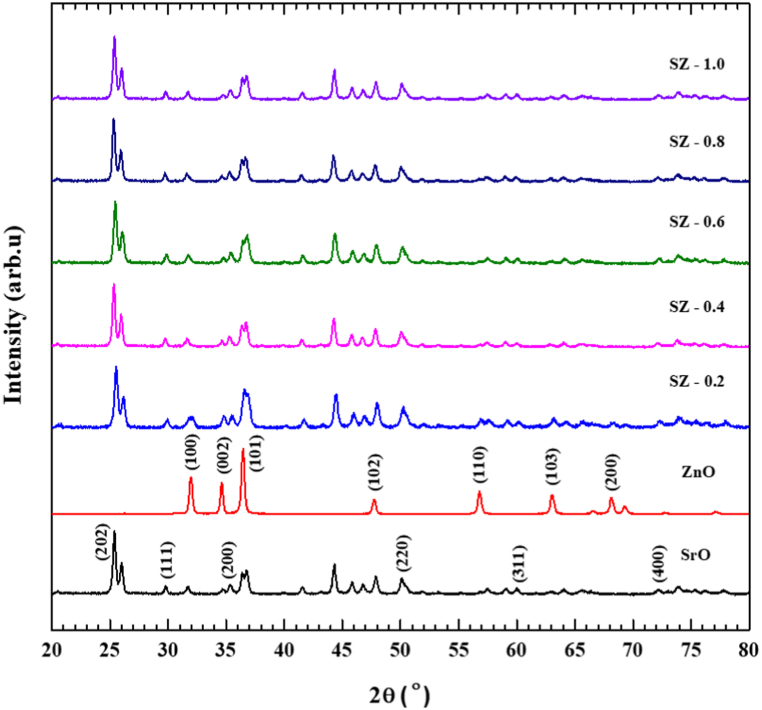
X-ray diffraction patterns of SrO–ZnO nanocomposites.

The XRD pattern of SrO, ZnO NPs, and SrO–ZnO NCs were shown in Fig. 2. Strong diffraction peaks observed at 2θ values of 25.38, 30.30, 35.13, 50.53 and 60.07° corresponds to SrO diffraction planes (202), (111), (200), (220), and (311), respectively belonging to Fm-3m space group (JCPDS no. 06–520) [[32], [33], [34]]. In the XRD pattern of ZnO NPs, the 2θ peaks were appeared at 31.73, 34.36, 36.20, 47.46, 56.52, 62.75, and, 66.29°, these peaks were corresponding to (100), (002), (101), (102), (110), (103) and (200) planes of ZnO, respectively. All of these XRD peaks were consistent with the standard diffraction data belonging to the hexagonal wurtzite phase of ZnO (JCPDS card no. 89–0511) [25,35,36]. In the XRD pattern of SrO–ZnO NCs, all characteristic peaks of SrO and ZnO were present. Though the inclusion of ZnO can be identified from the appearance of relevant peaks, there was predictable increase in the intensity of these peaks with the increase in the concentration of ZnO precursor opted during the synthesis of SrO–ZnO NCs. In all the NCs samples, only the diffraction peaks of SrO were dominant. From this observation, it can be inferred that SrO–ZnO NCs were not just a mixture of SrO and ZnO, instead, major portion of Zn may be doped within the crystalline lattice of SrO [23].

**Fig. 3:**
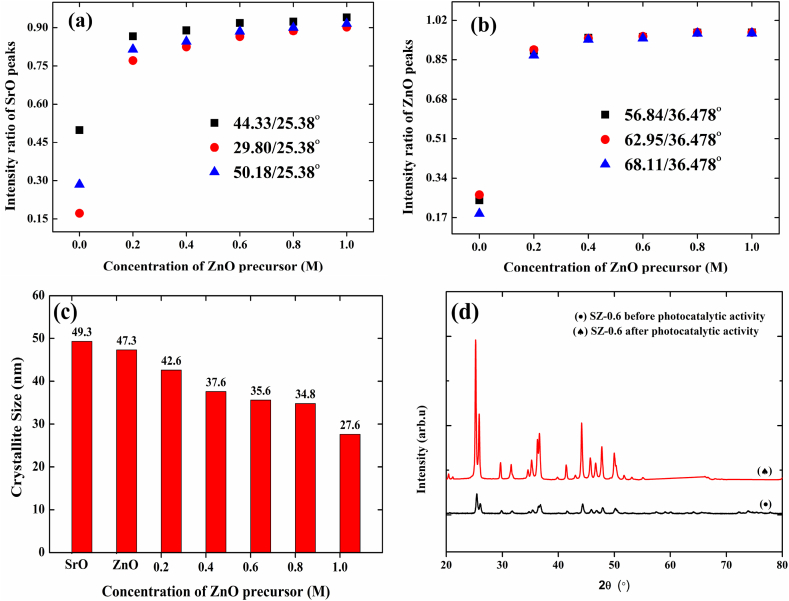
Changes in intensity of XRD peaks of SrO (a), ZnO (b), and grain size of crystals in SrO–ZnO nanocomposites (c) as a function of concentration of ZnO precursor, d) X-ray diffraction patterns of SrO–ZnO nanocomposites before and after photocatalytic activity.

In order to get further insight on this observation, all XRD patterns were subjected to statistical analysis. Based on the intensity of the major peak, 25.38° (202), the overall intensity of SrO and SrO–ZnO NCs were normalized unity. In the case of ZnO, the XRD pattern was normalized to unity on the basis of the intensity of the predominant peak, 36.47° (101). First the change in the crystalline structure of SrO with respect to the concentration of ZnO precursor, ratio of intensities of the major peak, and other clearly distinguishable peaks that appeared in between the ZnO peaks was taken into consideration. The 44.33°, 29.80°, and 50.18° peaks of SrO selected for comparison, as they were clearly distinguishable and not merged with ZnO peaks. The plots showing the changes in ratio intensities of these peaks, i.e., 44.33°/25.33°, 29.80°/25.33°, and 50.18°/25.33°, were illustrated in Fig. 3a. When compared with neat SrO, the intensity ratio of all these peaks in SZ-0.2 was 1.8–3.2 times higher. Further increase in the concentration of ZnO precursor had no significant effect. Only a slight increase in the ratios of peak intensity was noticed. Likewise, changes in the crystalline structure of ZnO were also assessed. The ratio of intensity of the major ZnO peak, 36.47°, and the other distinct ZnO peaks at 56.84°, 62.95°, and 68.11° were taken into consideration. As shown in Fig. 3b, the intensity ratio of 56.84°/36.47°, 62.95°/36.47°, and 68.11°/36.47° in neat SZ-0.2 was 3–5 times higher than ZnO. Similar to SrO, only a slight increase in the ratio of peak intensities was observed due to a further increase in the concentration of ZnO from 0.4 to 1.0 M. The observation gained from Fig. 3 a-b, confirms the dominance of SrO in the SZ NCs, irrespective of the concentration of ZnO precursor fed initially during synthesis, and both SrO and ZnO were slightly increased the crystallinity of each other. The average grain size of SrO, ZnO, and SrO–ZnO–NCs was estimated using Debye-Scherer's formula. The grain size estimated for SrO was 49.3 nm, and it was 47.3 nm for ZnO NPs. Fig. 3c shows changes in grain size with respect to increase in the concentration of ZnO precursor. The grain size was gradually decreased as with increase in the concentration of ZnO precursor. The gradual decrease in crystallite size also indicate that the incorporation of Zn^2+^ ions into Sr^2+^ lattice positions. Unlike, hydrothermally prepared SZ NCs prepared by Harish et al., here, no additional peaks emerged. SZ NCs prepared by Harish et al., were exhibited two additional peaks at 41.78 and 48° corresponding (211), and (204) planes of orthorhombic phase of ternary compound, SrZnO_2_ [25]. Therefore, XRD pattern of SZ NCs obtained here confirmed the occurrence of both doping of Zn^2+^ ions into Sr^2+^ lattice and formation of SrO–ZnO composite.

Fig. 3d shows the XRD spectra of 0.6 M SrO–ZnO NCs before and after photocatalytic experiment. The catalyst was collected after the photocatalytic experiment and subjected to the XRD analysis. The observed peaks of 0.6 M SrO–ZnO NCs after photocatalytic experiment are well matched with the SrO–ZnO NCs before photocatalytic experiment. Also, no other peaks or suppression of peaks is observed after degradation studies for the sample. This result clearly indicates the stability of prepared NCs [23].

### 3.2 Functional group analysis of SrO–ZnO nanocomposites

**Fig. 4:**
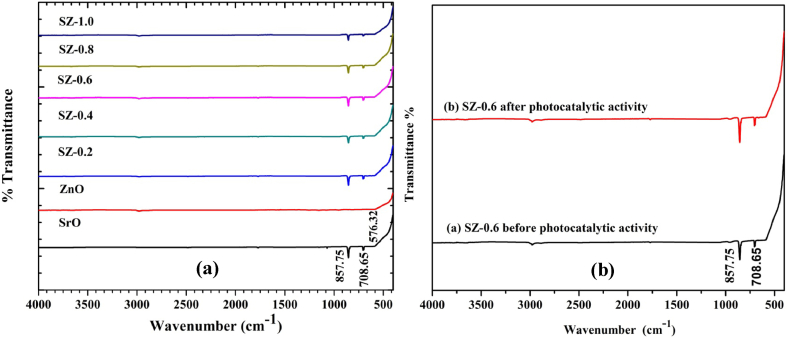
FT-IR spectra of a) SrO, ZnO and SrO–ZnO nanocomposites, b) FT-IR Spectra of 0.6 M SrO–ZnO nanocomposite before and after photocatalytic activity.

The chemical functional group characterization was done by scanning the prepared samples in the IR range 400–4000 cm^−1^. FT-IR spectra of SrO, ZnO NPs, and SrO–ZnO NCs are shown in Fig. 4a. The observed wavenumber from the recorded spectra and its corresponding assignments are demonstrated in the figure. In SrO and ZnO NPs, the band at 708 cm^−1^, 857 cm^−1^ and 1063 cm^−1^ are due to symmetric and asymmetric vibration of Sr–O bond, and the peak at 581 cm^−1^ shows Zn–O bending vibrations, respectively. For SrO–ZnO NCs, the peaks observed are well-matched with the bare SrO and ZnO confirming the presence of SrO and ZnO bonds in the composites samples. Moreover, there is no additional peaks found during FT-IR studies indicate the formation of desired samples. Also, Fig. 4 (b) shows the FT-IR spectra of 0.6 M SrO–ZnO NCS before and after photocatalytic activity measurements. The observed peaks for the 0.6 M SrO–ZnO NC after photocatalytic activity are well correlated with the peaks of the FT-IR spectrum of SrO–ZnO NC before activity. The absence of other peaks in the FT-IR spectrum of 0.6 M SrO–ZnO NC indicates that there are no residual belonging to MG dye is present in the sample. This result clearly indicates that the prepared NC strongly degrades the MG dye under sunlight. Also, the catalyst doesn't degrade after the activity which indicates the stability of the NC [29,31,37].

### 3.3 Thermal analysis (TG/DTA)

**Fig. 5:**
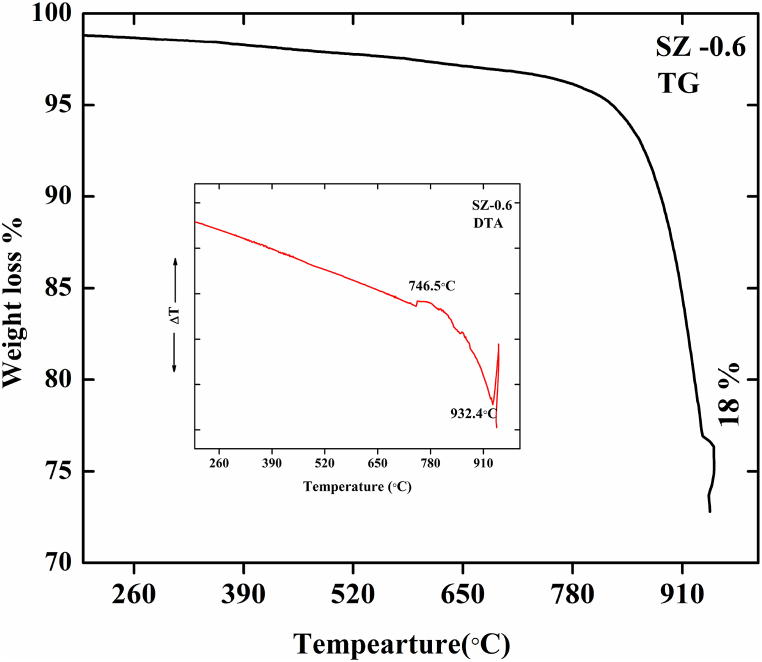
TG and DTA spectrum of 0.6 M SrO–ZnO nanocomposite.

Fig. 5 shows the TG and DTA curves of the 0.6 M SZ NCs sample obtained between 27 °C and 950 °C. Thermal decomposition was performed in dry air at the heating rate of 20 °C min^−1^. In the TG curve, it can be observed that there is a weight loss observed after 600 °C indicates that there is a thermal decomposition of carbonates present in the sample. An exothermic peak was observed in the DTA curve between 746.9 °C and 948.4 °C corresponding to the thermal decomposition of SrCO_3_. Also, it is observed that the decomposition was observed in the temperature above 600 °C with a weight loss of 18 % indicating the removal of surface impurities. The results revealed good thermal stability of SZ - 0.6 NCs [30,38,39].

### 3.4 Optical properties of SrO–ZnO nanocomposites

**Fig. 6:**
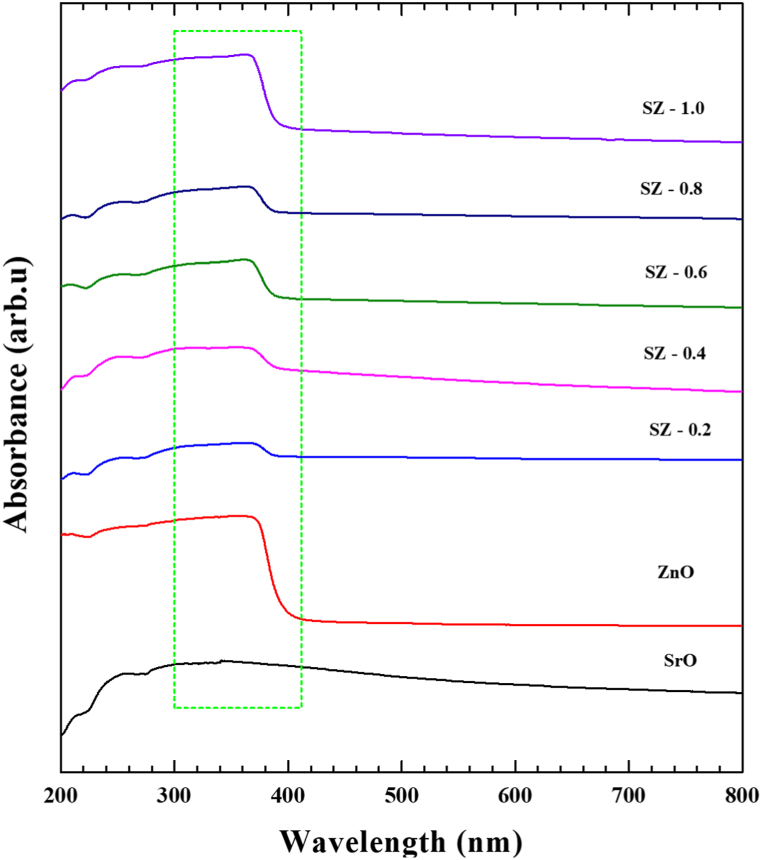
UV–Vis spectra of SrO–ZnO nanocomposites.

The UV–Vis absorption spectra of SrO, ZnO, and SZ NCs were shown in Fig. 6. The SrO exhibited broad absorption starting from UV to the visible region (700 nm) with a peak at 340 nm. In ZnO, the entire absorption range was well within the UV region. Almost, there was no light absorption above 400 nm. Based on the concentration of ZnO precursor, the nature of light absorption of SZ NCs were varied between SrO to ZnO. The electronic absorption of SZ-0.2 was mostly similar to SrO. Upon increasing the concentration of ZnO, the absorption was gradually transformed. In SZ-1.0, the nature of absorption range and peak become almost identical to neat ZnO. In contrary to the XRD pattern, where SrO peaks were dominated in SZ NCs, UV–Vis spectra of SZ, a clear influence of ZnO was witnessed. Upon increase in the concentration of ZnO precursor, the UV–Vis spectra of SZ NCs were getting predominantly occupied by the electronic absorption peak of ZnO. Here it is very important to note that UV–Vis spectra were measured in the diffuse reflectance method. In the diffuse reflectance mode, the spectra were obtained as result of incident radiation directly reflected from the material's surface and also reflected after diffusing in to the materials [24]. As light with the wavelength spanning from near UV through visible to infrared regions, the photons interact with the first 10–20 nm of materials that can strongly absorb theses photons, such as semiconductors, alloy and metals. In case of non-transparent materials, these photons can interact with 50–100 atomic layers [40]. When analyze the obtained UV–Vis spectra of SZ NCs, by sticking with the principle of diffuse reflectance method, it can be understood that some part of ZnO was coated on the surface of SrO. XRD patterns of SZ NCs also indicated that a portion of ZnO precursor lead to the formation of ZnO coating on SrO.

**Fig. 7:**
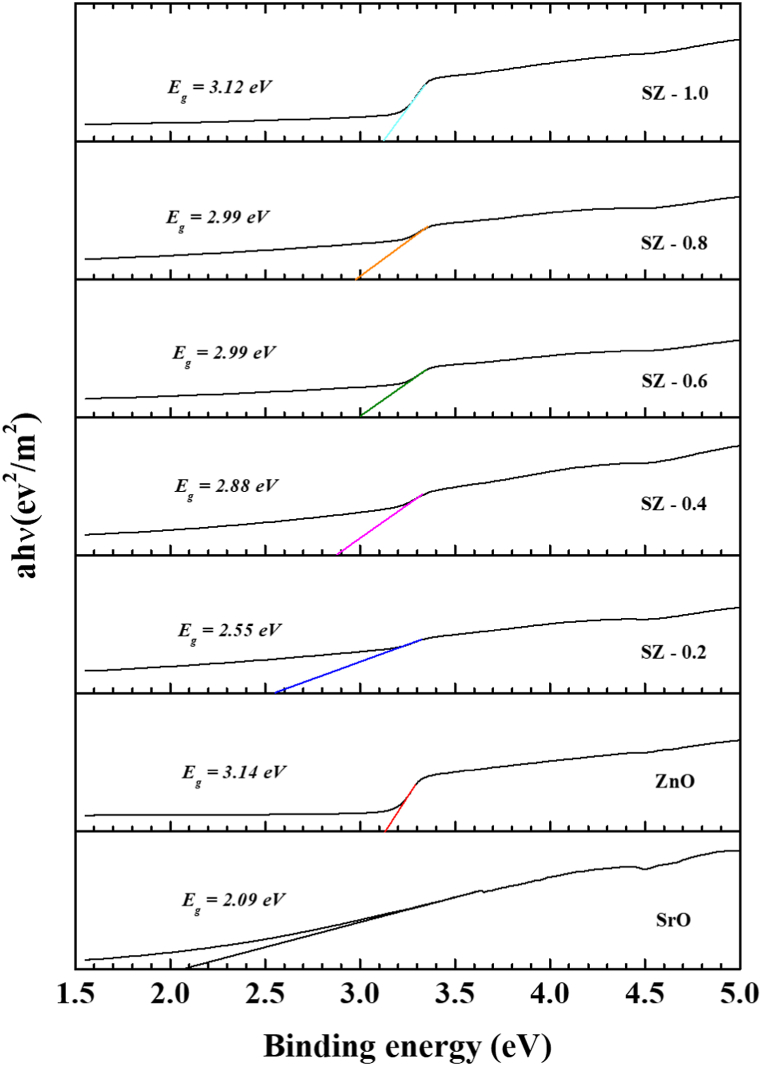
Tuac plot of SrO–ZnO nanocomposites.

**Fig. 8:**
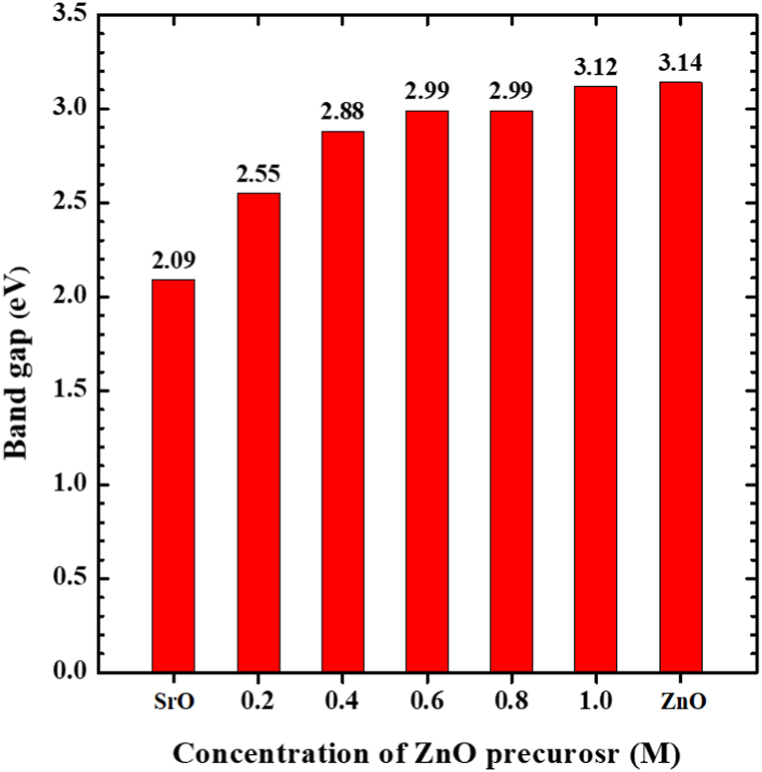
Changes in optical band gap of SrO–ZnO nanocomposites as a function of concentration of ZnO precursor.

The optical band gap of SrO, ZnO, and SZ NCs were estimated by using Tauc – Lorentz relation: (α hν)^n^ = B(hν-E_g_), where, α = absorbance, hν = photon energy, *E*_g_ = optical band gap, B = constant relevant to the material, n = 2 for direct transition, and n = 1/2 for indirect transition. The *E*_g_ can be obtained by extrapolating the linear portion of (α hν)^n^ curve to zero. Fig. 7 show the band gap diagram of SrO, ZnO and SZ NCs. The changes in *E*_g_ of SZ NCs as a function of increasing concentration of Zn precursor was shown in Fig. 8. The *E*_g_ of neat SrO was 2.09 eV, and it was increased to 2.55 eV for SZ-0.2. For SZ-0.4, SZ-0.6 and SZ-0.8, the band gap increased to 2.88, 2.99 and 2.99 eV, respectively. The E_g_ of SZ-1.0 (3.12 eV) was almost become equal to E_g_ of neat ZnO (3.14 eV). The trend observed in E_g_ of SrO, ZnO and SZ NCs were consistent with their UV–Vis spectra, where with increase in the concentration of ZnO precursor, the range of electronic absorption of SZ NCs were gradually become identical to ZnO. Similarly, the same trend was repeated in *E*_*g*_ of SZ-1.0 and ZnO. In the literature, the SrO NPs (*E*_*g*_ = 2.2 eV) and nanorods (*E*g = 2.4 eV) synthesized using wet chemical method were exhibited the broad absorption with the peak 320, and 297 nm respectively [22,23]. The E_g_ and electronic absorption was comparable with these reports. The obtained E_g_ value of SrO was just above the value reported for bulk SrO (Eg = 1.8 eV) [24]. The absorption at UV region and *E*_*g*_ of ZnO synthesized using wet chemical was well within the results reported in relevant literature [41,42].

### 3.5 Morphology and elemental composition of SrO–ZnO nanocomposites

**Fig. 9:**
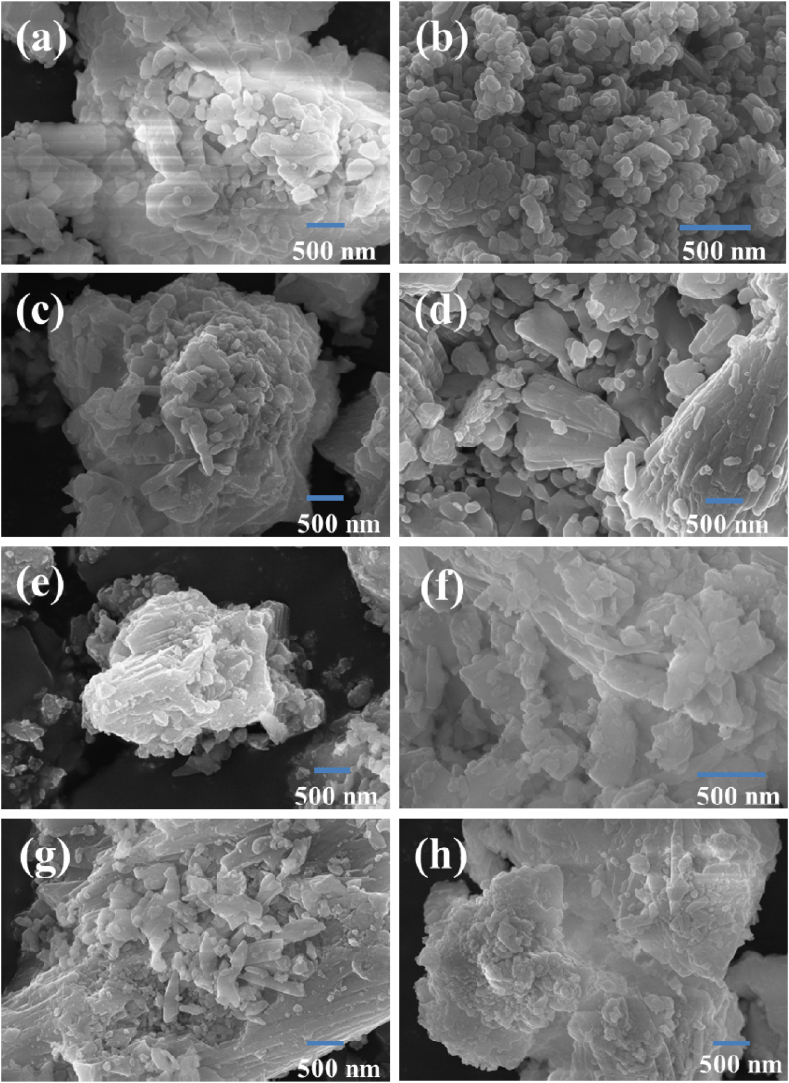
FESEM images of SrO–ZnO nanocomposites: (a) SrO, (b) ZnO, (c) SZ-0.2, (d) SZ-0.4, (e) SZ-0.6 – Zone 1, (f) SZ-0.6 – Zone 2, (g) SZ-0.8, and (h) SZ-1.0.

**Fig. 10:**
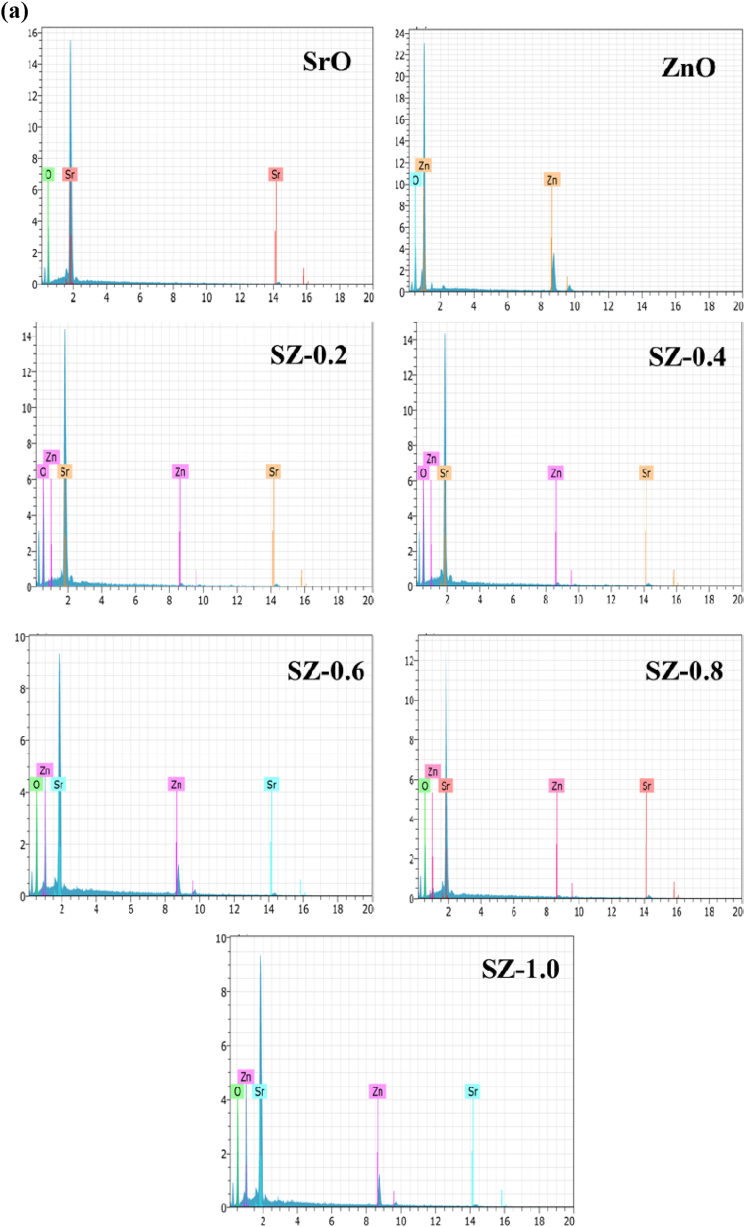

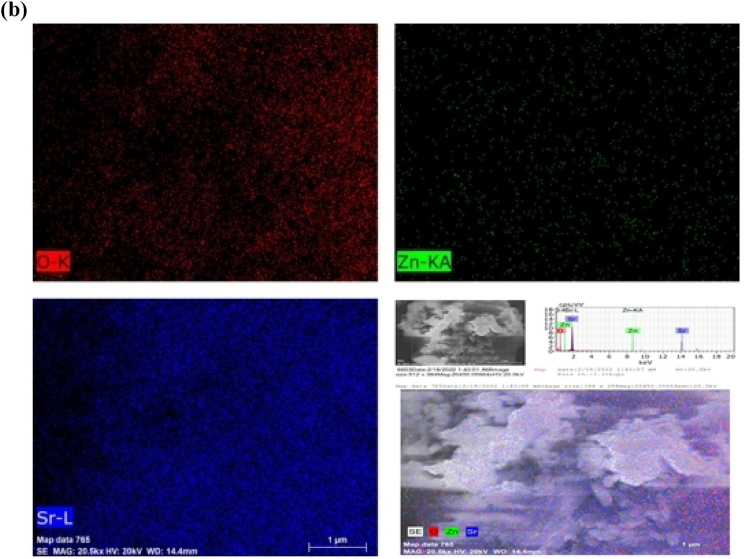
**(a).** EDS spectra of SrO–ZnO nanocomposites. **(b).** EDS mapping of Sr, Zn, and O elements.

Fig. 9 (a-h) shows the FESEM images of SrO, ZnO and SZ NCs. In SrO, on a cluster, the sub-100 nm nanostructures with varying shapes such as spherical, cube, random and rod-like, were seen. Whereas, in ZnO uniform semi-cylindrical domains varying in diameter and length were observed. SZ-0.2 exhibited layered structure, on which spherical, cylindrical and rod-like domains were spotted. In SZ-0.4 and SZ-0.6, a cluster of nanostructures with different shapes was observed. When closely observed, planer and random shaped nanostructures were found in SZ-0.6. In SZ-0.8, a mixture of rod-like, random and sub 50 nm spherical domains can be located on the well grown layered structure. In SZ-10, a layered cluster having spherical shaped domain coated on the surface was seen. The appearance of heterostructures on the SZ NCs confirms the formation SrO and ZnO composite and doping of Zn in the SrO lattice. Fig. 10 (a) shows the EDS spectra of SrO, ZnO NPs and SZ NCs. SrO and ZnO contain Sr + O and Zn + O peaks, respectively. In SZ NCs, strontium, oxygen and Zinc atoms were present and intensity of Sr peak was decreased with increasing the concentration of ZnO precursor. The Sr content was decreased due to the addition of Zn^2+^ in the strontium sites [31]. The raw EDS mapping of various elements present in the sample is shown in Fig. 10 (b). Different elements present in the sample are indicated by a different color and the selected area for the EDS scanning shows the uniform distribution of elements in the entire area. This adds further support for the above 10.13039/100004679EDS spectra analysis. The atomic and weight percentage of prepared samples are shown in Table 1.

**Table 1:** Elemental composition of SrO, ZnO and SZ NCs.

Samples	Element	Normal %	Atomic %
**SrO**	Sr O	59.95 40.05	21.47 78.53
**ZnO**	Zn O	64.19 35.81	30.49 69.51
**SZ-0.2 M**	Sr O Zn	59.44 40.50 0.06	21.18 78.84 0.03
**SZ-0.4 M**	Sr O Zn	51.66 47.03 1.11	16.61 82.82 0.55
**SZ-0.6 M**	Sr O Zn	60.73 38.16 1.31	22.39 77.06 0.57
**SZ-0.8 M**	Sr O Zn	49.94 38.95 11.10	17.96 76.70 5.35
**SZ-1.0 M**	Sr O Zn	41.18 44.03 14.79	13.63 79.81 6.56

### 3.6 Photocatalysis degradation of malachite green dye by SrO–ZnO nanocomposites

**Fig. 11:**
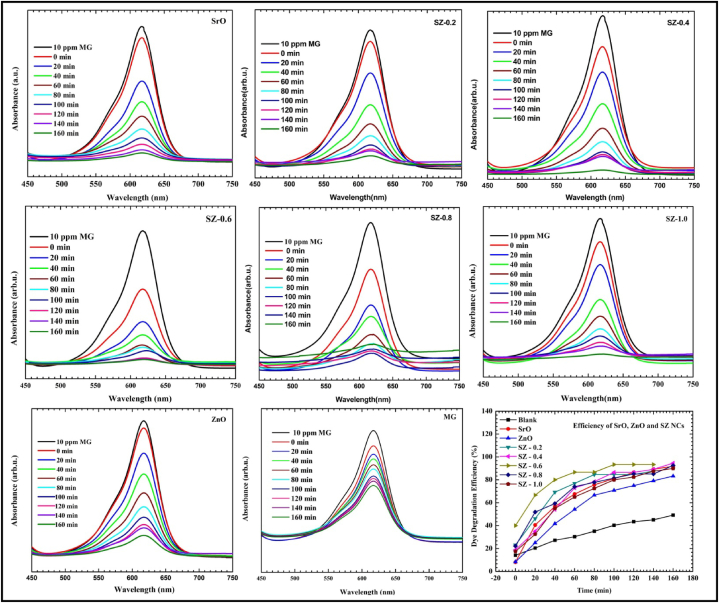
Photocatalytic properties of SrO-ZNO nanocomposites; UV–Vis spectra of Malachite Green dye as a function of photocatalysis time and overall photocatalytic efficiency. (For interpretation of the references to color in this figure legend, the reader is referred to the Web version of this article.)

Fig. 11 shows the UV–Vis spectra of MG dye treated with SrO, ZnO NPs and SZ NCs, and the overall photocatalytic efficiency of these materials. As a function of time, the characteristic absorbance of MG at 617 nm was decreased gradually. The MG solution being treated became colorless after 160 min. When compared with ZnO, both SrO and SZ NCs were led to the fast decolorization of MG. The MG decolorization was attributed to the non-selective photooxidation caused by reactive oxygen radicals including, hydroxyl and superoxide radicals upon exposure to direct sunlight. The SrO and ZnO did not adsorb MG, whereas the SZ-0.2, SZ-0.4, SZ-0.8 and SZ-1.0 were adsorbed almost 15 % of MG. Though there were a few % changes in the amount of MG removed after 160 min, the rate of MG removal has appeared the same. SZ-0.6 was adsorbed almost 30 % of MG and led to rapid MG removal. ZnO exhibited the lowest efficiency due to poor solar light absorption. The amount of MG degraded with SrO and ZnO was 86 % and 83 %, respectively. The % of MG degradation efficiency estimated for SZ NCs are as follows: SZ-0.2 = 93; SZ-0.4 = 97; SZ-0.6 = 98; SZ-0.8 = 94; and SZ-1.0 = 92 %. Among different composites, the Sr–Zn-0.6 has the highest photocatalytic activity and completely degrades the Malachite green within 140 min under direct sunlight. The highest photocatalytic efficiency of SZ NCs was due to the ease of photoactivation in direct sunlight resulting from the presence of both SrO and ZnO.

**Fig. 12:**
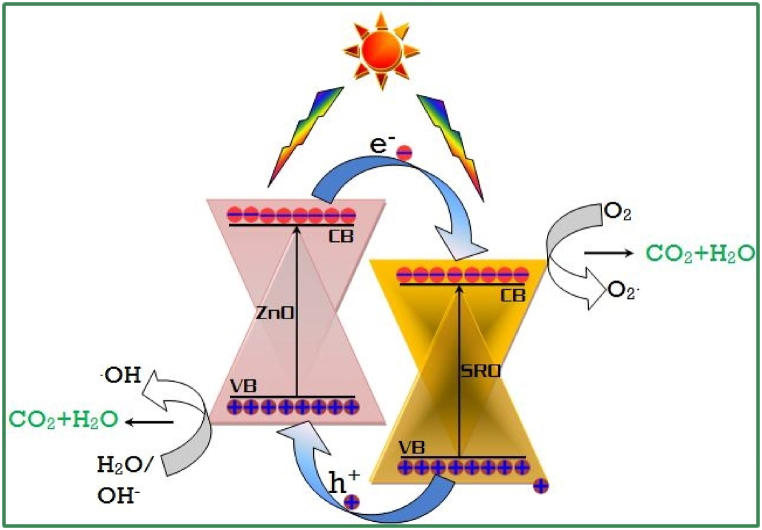
Photocatalytic mechanism of Hybrid SrO–ZnO NCs.

The above results showed that the hybrid photocatalyst are most efficient than single one. In the case of NCs, the valence band (VB) of ZnO is located in between the conduction (CB) and VB of SrO, and the CB of ZnO is located above the VB and CB of SrO. Whenever the light radiation incident on the photocatalyst, the electrons from the valence band of ZnO is transferred to the conduction band of ZnO. As time passes, the electrons in the conduction band of ZnO are transferred to the conduction band of SrO as it locates just below the conduction band of ZnO. Also, the holes are transferred from the valence band SrO to the valence band of ZnO. This transfer causes the effective charge separation and suppression of carrier recombination in NCs. As a result, there will be higher production of O_2_^•-^ and ^•^OH radicals [[43], [44], [45], [46], [47], [48], [49], [50]], facilitating to the rapid oxidation of pollutants. The proposed photocatalytic mechanism is shown in Fig. 12.

**Fig. 13:**
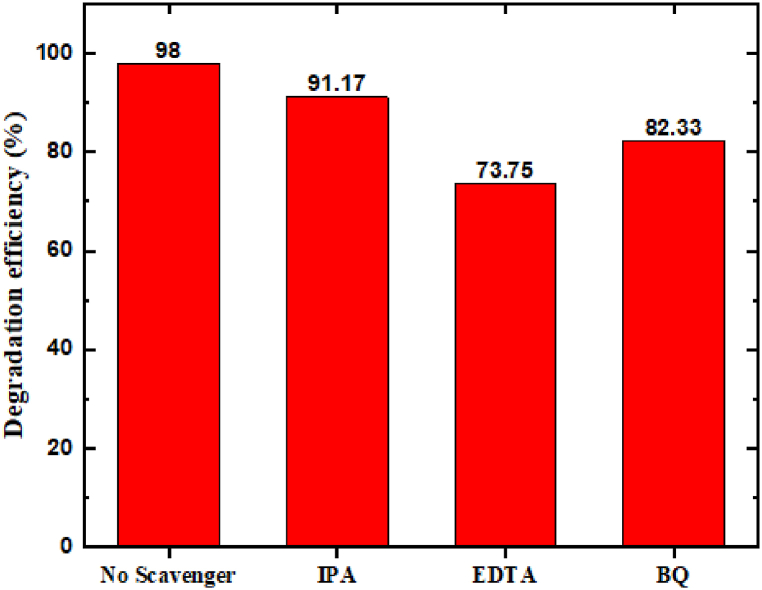
Trapping experiment of MG using Hybrid SrO–ZnO NCs.

Based on the above results obtained, we propose a possible Photocatalytic mechanism of the degradation of MG under visible-light irradiation

(1) SrO / ZnO + hv → SrO /ZnO (e^−^/h^+^)

(2) O_2_ + e− → •O^2−^

(3) H_2_O + h+ → •OH + h^+^

(4) •O_2_^−^ + 2H + e^−^ → H_2_O_2_

(5) H_2_O_2_ + e^−^ → •OH + OH^−^

(6) h^+^ + OH^−^ → •OH

(7) •OH + MG → CO_2_ + H_2_O + NO_3_^−^ + NH_4_++ Cl^−^

Furthermore, the radical scavenger experiment of the active species during the sonocatalytic process is shown in Fig. 13. The scavengers such as IPA, EDTA, and BQ, were used as hydroxy radicals (.OH), holes (h^+^), and superoxide radicals (.O_2_^−^), respectively [[51], [52], [53], [54], [55], [56]]. The photocatalytic efficiency of SrO–ZnO NCs using IPA, EDTA, and BQ were, 91.17 %, 73.75 %, and 82.33 %, respectively. These results revealed that.O_2_^−^, and.OH are the major active species in MG pollutant degradation using SrO–ZnO NCs.

**Fig. 14:**
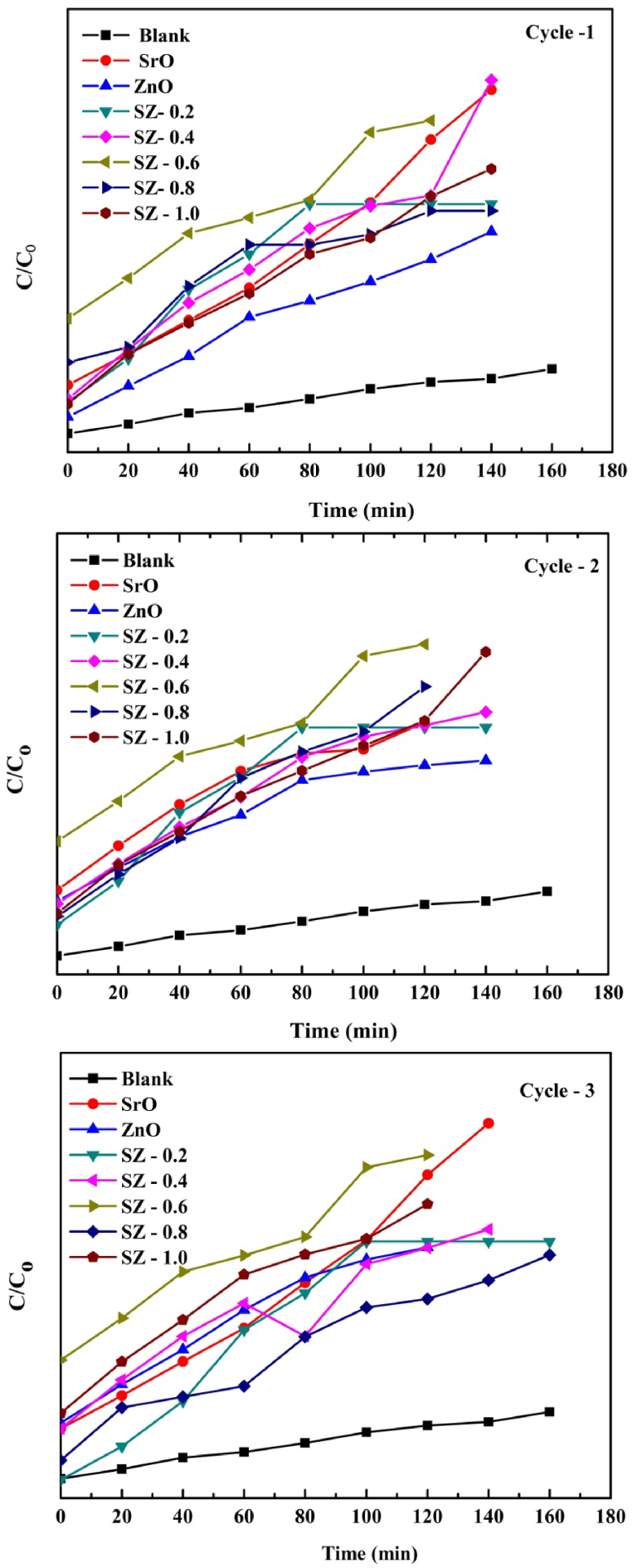
Photocatalytic data of first (experiment), second and third run.

Fig. 14 shows the photocatalysis experiment data of first (experiment), second and third run. The graph is plotted time against C/C_0_. From the third run, we do not observe much variation. The result indicates the repeatability and stability of the prepared sample.

The pseudo–first-order reaction [23] through a Langmuir – Hinshelwood (LH) rule was used to measure the degradation of dyes as follows.

In (C_0_ /C) =Kt

Where, C_0_ = Initial absorbance, C = Absorbance after a time, K = First - order rate constant, t = time of irradiation.

**Fig. 15:**
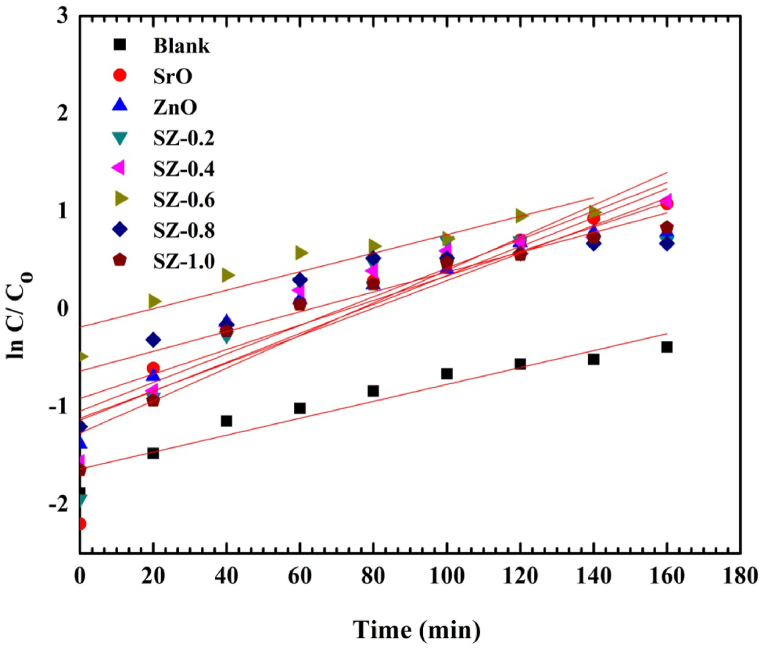
Pseudo-first-order kinetic plot for photo-decolorization of MG solution.

The obtained photocatalytic data were found to be fit with pseudo –first – order kinetic model in which ln (C_0_/C) was plotted for all the reactions against the reaction time (min). Fig. 15 shows the linear relationships plot of ln (C_0_/C) vs irradiation time. Straight lines were obtained with correlation co-efficient R^2^ and the slope represents Rate constant K (min^−1^) is known as pseudo-first-order reaction. The obtained kinetic data confirms the higher reaction associated for 0.6 M SZ NCs in the prepared samples. The obtained kinetic parameters are tabulated in Table 2. The obtained result for the synthesized sample is compared with standard references and is tabulated in Table 3.

**Table 2:** Pseudo first order kinetic Parameters.

Samples	Rate constant K (min^−1^)	Correction Coefficient (R_2_)
SrO	0.01668	0.89978
ZnO	0.01254	0.86717
SZ-0.2 M	0.0148	0.72551
SZ-0.4 M	0.09751	0.86308
SZ-0.6 M	0.00946	0.87146
SZ-0.8 M	0.04012	0.74661
SZ-1.0 M	0.00265	0.85320

**Table 3:** Comparison of MG degradation efficiency of SZ NCs with other reference photocatalyst.

Catalyst	Catalyst amount	Dyes	Light Source	Degradation % time	Reference
ZnO -SrO	0.1 mg/50 ppm	MG	UV lamp	220 min	[52]
SrO–SnO_2_	0.35 mM, 0.75 mM	Azo dye, pesticide	UV lamp	–	[53]
Ag_2_O. SrO. CaO	0.03 g L^−1^, 0.04 g L^−1^, 0.05 g L^−1^, 0.06 g L^−1^, 0.07 g L^−1^	MV dye	Visible light	Ag_2_O = 66/120 min SrO = 72/120 min CaO = 43/120 Ag_2_O. SrO. CaO = 100/120	[54]
SrO/GO	0–80 mg L^−1^	100 mg L^−1^	Solar light	74.5	[55]
Sr doped ZnO	10 mg L^−1^	MB	Xenon lamp	50/45min	[56]
SrO–ZnO	10 ppm	MB	Visible light	50/120 min	[22]
\SrO–ZnO	1mg/10 ppm	MG	Visible light	98**/**160 min	Present work

## 4 Conclusion

SZ NCs were synthesized by the co-precipitation method. The XRD patterns and UV spectra confirmed the formation of SZ NCs. A major portion of Zn atom was doped in the SrO lattice and remaining were coated as ZnO on the surface of SrO. The presence of SrO extended the light absorption of SZ NCs to the visible region. The incorporation of ZnO increased the bandgap of SZ NCs from 2.09 to 3.12, depending on the concentration of ZnO precursor used during synthesis. The sub-100 nm-sized heterostructures were found in the FESEM images of SZ NCs. The elemental composition estimated by EDS spectra also confirmed the presence of Sr, Zn, and O in the SZ NCs. The SZ-0.6 sample exhibited the highest photocatalytic efficiency under direct sunlight. The combination of SrO and ZnO were favored the effective use of direct sunlight for environmental photocatalysis. Aforementioned finding suggests that SZ-0.6 NC sample can be a safe and potential sunlight photocatalyst useful for practical and cost-effective water purification. As SZ NCs were proved to be activated by direct sunlight, they can be used for water purification in remote areas without electricity.

## Declaration of interest's statement

The authors declare no conflict of interest.

## Data availability statement

Data will be made available on request.

## Additional information

No additional information is available for this paper.

## CRediT authorship contribution statement

**Govindharaj Anandhakumari:** Conceptualization, Data curation, Writing – original draft. **Palanisamy Jayabal:** Data curation, Formal analysis, Methodology, Writing – original draft. **Athinarayanan Balasankar:** Data curation, Formal analysis, Investigation, Writing – original draft. **Subramaniyan Ramasundaram:** Data curation, Formal analysis, Software, Validation. **Tae Hwan Oh:** Formal analysis, Resources, Validation, Writing – review & editing. **Kanakaraj Aruchamy:** Formal analysis, Resources, Software. **Parashuram Kallem:** Formal analysis, Resources, Writing – review & editing. **Veerababu Polisetti:** Formal analysis, Resources, Writing – review & editing.

## Declaration of competing interest

The authors declare that they have no known competing financial interests or personal relationships that could have appeared to influence the work reported in this paper.
